# Astragaloside IV Ameliorates Isoprenaline-Induced Cardiac Fibrosis in Mice *via* Modulating Gut Microbiota and Fecal Metabolites

**DOI:** 10.3389/fcimb.2022.836150

**Published:** 2022-05-17

**Authors:** Xu-Qin Du, Li-Peng Shi, Zhi-Wei Chen, Jin-Yuan Hu, Biao Zuo, Yu Xiong, Wen-Fu Cao

**Affiliations:** ^1^ College of Traditional Chinese Medicine, Chongqing Medical University, Chongqing, China; ^2^ Chongqing Key Laboratory of Traditional Chinese Medicine for Prevention and Cure of Metabolic Diseases, Chongqing Medical University, Chongqing, China; ^3^ Department of Chinese Traditional Medicine, The First Affiliated Hospital of Chongqing Medical University, Chongqing, China

**Keywords:** Astragaloside IV, cardiac fibrosis, gut microbiota, fecal metabolites, Akkermansia

## Abstract

**Aim:**

Gut microbiota is of crucial importance to cardiac health. Astragaloside IV (AS-IV) is a main active ingredient of Huangqi, a traditional edible and medicinal herb that has been shown to have beneficial effects on cardiac fibrosis (CF). However, it is still uncertain whether the consumption of AS-IV alleviates cardiac fibrosis through the gut microbiota and its metabolites. Therefore, we assessed whether the anti-fibrosis effect of AS-IV is associated with changes in intestinal microbiota and fecal metabolites and if so, whether some specific gut microbes are conducive to the benefits of AS-IV.

**Methods:**

Male C57BL-6J mice were subcutaneously injected with isoprenaline (ISO) to induce cardiac fibrosis. AS-IV was administered to mice by gavage for 14 days. The effects of AS-IV on cardiac function, myocardial enzyme, cardiac weight index (CWI), and histopathology of ISO-induced CF mice were investigated. Moreover, 16S rRNA sequencing was used to establish gut-microbiota profiles. Fecal-metabolites profiles were established using the liquid chromatograph-mass spectrometry (LC-MS).

**Results:**

AS-IV treatment prevented cardiac dysfunction, ameliorated myocardial damage, histopathological changes, and cardiac fibrosis induced by ISO. AS-IV consumption increased the richness of Akkermansia, Defluviitaleaceae_UCG-011, and Rikenella. AS-IV also modulated gut metabolites in their feces. Among 141 altered gut metabolites, amino acid production was sharply changed. Furthermore, noticeable correlations were found between several specific gut microbes and altered fecal metabolites.

**Conclusions:**

An increase of Akkermansia, Defluviitaleaceae_UCG-011, and Rikenella abundance, and modulation of amino acid metabolism, may contribute to the anti-fibrosis and cardiac protective effects of Astragaloside IV.

## Introduction

Cardiac fibrosis (CF) is characterized by cardiac fibroblasts hyperproliferation and extracellular matrix accumulation, which ultimately becomes an irreversible contributor to heart failure (HF) (Travers et al., 2016; Wu, 2021). CF is an inevitable pathological process of HF. However, no effective drug for CF has been developed. Since the potential mechanism of CF is not fully understood, discovering novel mechanisms, and identifying underlying therapeutic targets are of vital importance for effective anti-fibrosis intervention and delaying HF.

In recent years, the contributory role of gut microbiota and its metabolic products on host cardiac health has been revolutionized understanding through the application of high-throughput sequencing technology and metabolomics analysis (Lee and Hase, 2014; Miele et al., 2015; Du et al., 2020). It has been recognized that gut microbes are a vital role in cardiac fibrosis (Karbach et al., 2016; Organ et al., 2016). Numerous gut microbiota and fecal metabolites have been identified, causing interactions between microbiota-host and metabolic pathways connecting the gut and CF (Li et al., 2019; Du et al., 2020; An et al., 2021). Nonetheless, the effect of gut microbiota and its metabolites on CF remains elusive. An in-depth study on the features of CF-related gut microbiota and metabolites might provide a new and original therapeutic strategy that ameliorates cardiac fibrosis and delays the transformation from CF into HF.

Astragaloside IV (AS-IV) is a main active ingredient extracted from Huangqi, a traditional edible and medicinal herb that has been used for over 2000 years (Ren et al., 2013). Previous evidence suggested that Astragaloside IV exerts diverse biological activities, such as anti-oxidation (Luo et al., 2021), suppressing inflammation (Wan et al., 2018), modulating gut microbiota and gut-derived metabolites (He Q. et al., 2020; Gong et al., 2021). AS-IV has also shown notable pharmacological effects on anti-myocardial fibrosis (Lin et al., 2019; Wei et al., 2020). Although the cardioprotective effects of AS-IV have been proved, whether modulation of intestine microbes and metabolic products by AS-IV reduces isoprenaline-induced cardiac fibrosis has not been investigated.

Herein, we examined the adverse effects on gut microbiome and metabolites in isoprenaline-induced CF mice, and investigated whether modulating the intestine microbes and metabolites by AS-IV ameliorated isoprenaline-induced cardiac damage and fibrosis.

## Materials and Methods

### Experimental Animals

C57BL-6J male mice (18~22g) were provided by the Department of Laboratory Animal Center, Chongqing Medical University (Chongqing, China). All experimental mice were kept in a specific pathogen-free laboratory animal center (SYXK 2018-0003) under a standard 12h light-dark cycle. Experimental animal protocols were approved by the Animal Experiments Ethical Review Committee of Chongqing Medical University (Chongqing, China) under No. 2021040.

### Experimental Design

**Figure 1:**
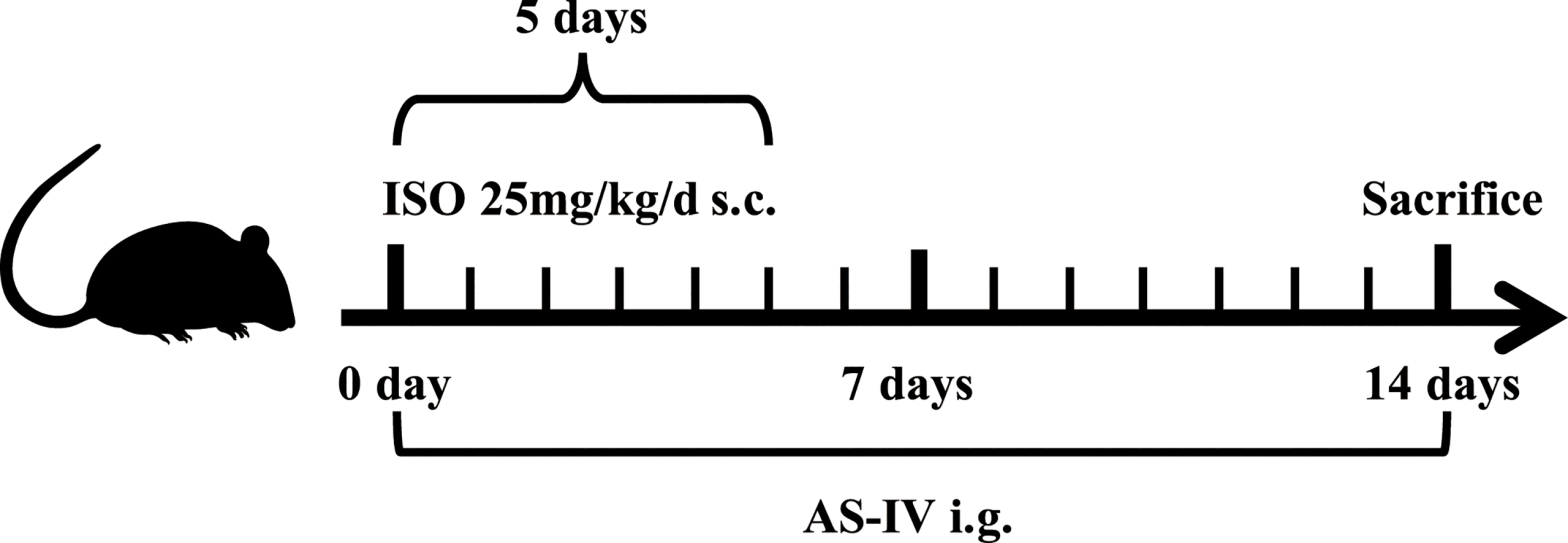
Timeline of the experimental design.

**Figure 1** shows the timeline of this animal experimental design. All mice were randomly allotted into the following three groups: control (n=15), model (n=20), and AS-IV (n=20). Mice in the AS-IV groups were orally administered AS-IV (100 mg/kg/d) for 14 days (Wan et al., 2018). AS-IV (HPLC purity> 98%) was bought from Chengdu Chroma-Biotechnology Co., Ltd., Chengdu, China, and dissolved in carboxymethyl cellulose (CMC) aqueous solution (0.5%). Mice in the remaining two groups were given 0.5% CMC aqueous solution by gavage. Concurrently, mice in the model and AS-IV groups received subcutaneous isoprenaline (ISO) (25 mg/kg, Sigma, USA) injection once a day for 5 days and then were left for a further 9 days to develop cardiac fibrosis (Samuel et al., 2014; Cáceres et al., 2019), while the control group was injected with 0.9% sterile saline. On the 14th day of the experiments, feces samples were collected aseptically and stored at -80°C until further processing. After the experiments, the animals were weighed. Subsequently, echocardiography detection was performed in mice anesthetized with isoflurane. The blood was immediately collected, the heart tissue samples were isolated, and their heart weights were measured.

### Echocardiography Assessment

After 2 weeks of experimental intervention, the mice were anesthetized with 3.5% inhaled isoflurane, while the continuous application of anesthesia was dropped to 1.5% isoflurane. The left ventricular contractile functional parameters of the mice, including fractional shortening (FS) and ejection fraction (EF), were measured noninvasively by transthoracic echocardiography using M-mode images from the parasternal long-axis equipped with a 23 MHz probe (6LAB; VINNO, Suzhou, China).

### Measurements of Plasma Biochemical Parameters and Heart Mass

The serum levels of myocardial enzyme, including creatine kinase (CK) and lactate dehydrogenase (LDH), were analyzed (n=10 mice per group) by using kits with an automatic biochemical analyzer (Chemray 800, Rayto, Shenzhen, China). After blood collection, mice were euthanized by 5% isoflurane anesthetics and 100% CO2 concentrations (Marquardt et al., 2018). Then the heart tissues were immediately excised and weighed. The cardiac weight index (CWI) was obtained by dividing heart weight (HW, mg) by body weight (BW, g).

### Histopathological Examination

The heart tissues used for histopathological examination were fixed in 4% paraformaldehyde for 24h. Subsequently, the fixed heart tissues were paraffin-embedded and then sectioned (5 μm) for hematoxylin-eosin (HE) and Masson’s trichrome (Masson) staining. HE and Masson staining were used to evaluate cardiac morphology and collagen deposition, respectively. The results were evaluated using a light microscopy, and five positive areas selected randomly from each stained section were photoimaged (magnification ×400). The stained signals were analyzed using the Image-Pro Plus (Bethesda, MD, USA).

### Immunohistochemical Staining

The paraffin-embedded heart tissue sections (5 μm) were dewaxed, rehydrated, placed in citrate (PH6.0) antigen retrieval buffer, and then incubated with 3% H2O2 at room temperature for 25 min. The sections were blocked with serum. Thereafter, they were incubated overnight with anti-α-SMA (Servicebio, GB111364, China, 1:1000) at 4°C and then incubated with a secondary antibody of goat anti-rabbit IgG HRP-conjugated (Servicebio, GB23303, China, 1:200) at room temperature for 50 min. Subsequently, they were stained with diaminobenzidine (DAB) reagent (Servicebio, G1211, China), subjected to hematoxylin counterstaining, dehydrated, sealed, and followed by observation using a light microscopy. Five images (magnification ×400) per heart were recorded randomly and quantified with Image-Pro Plus (Version6.0).

### 16S rRNA Gene Sequencing

Total DNA was extracted from stool samples using the soil DNA kit. The V3-V4 variable region of 16S rRNA gene was amplified with the primers. PCR reactions were performed under the following cycling conditions: 95 °C for 3min and subjected to 27 cycles (95°C for 30s, 55°C for 30s, 72°C for 30s), and 72°C for 10min. Thereafter, the PCR products were extracted, purified, quantified, and paired-end sequenced with the MiSeq platform (PE300, Illumina, USA).

The raw sequencing sequences were conducted quality control using the Fastp software and assembled by Flash software simultaneously. Analyses of the valid data were carried out on the Majorbio Cloud Platform (www.majorbio.com). The relative abundance of microbe community was analyzed by bar plot at the phylum and genus levels. The Bray-Curtis distance algorithm was used for microbe β-Diversity estimation and visualization was conducted through principal coordinate analysis (PCoA). The significantly different taxa between the three groups were identified by effect size measurements (LEfSe) and linear discriminant analysis (LDA), with the LDA threshold of 4.0 and *p* < 0.05 (Zhu et al., 2018).

### Fecal-Metabolomics Analysis

The thawed stool samples were homogenized in methanol-water (4:1, vol/vol), subjected to sonication at 5°C for 30 min, incubation at -20°C for 30 min, and then centrifugation at 13,000g for 15min at 4°C. The supernatant of each sample was analyzed by the liquid chromatograph-mass spectrometry (LC-MS) instrument, equipped with a UPLC-TripleTOF system (AB Sciex, USA).

The Progenesis QI software (Version 2.3, Waters Corporation, Milford, USA) was used for processing LC-MS raw data. The filtered data of positive and negative modes were combined and exported for analysis. Orthogonal partial least-squares discriminant analysis (OPLS-DA) was used for detecting the metabolic variation between groups. Parameters (*P* < 0.05 and VIP value >1) generated from the OPLS-DA analysis were set to screen and identify differentially expressed metabolites. The different clusters of significantly altered metabolites were identified by a heatmap profile. The differential metabolites were mapped to pathways following the KEGG database.

### Statistical Analysis

SPSS software (Version22.0, IBM, USA) was adopted for statistical analysis. The results were shown as mean ± standard deviation (SD). Multi-group comparisons were conducted by one-way ANOVA followed by LSD or Dunnett’s T3 test. Correlations were recognized by Spearman correlation analysis. The significance was set at *P* < 0.05. GraphPad Prism software (Version9.3.1, CA, USA) was used for generating graphs.

## Results

### AS-IV Increased the Survival Rate of ISO-Induced Mice

**Figure 2:**
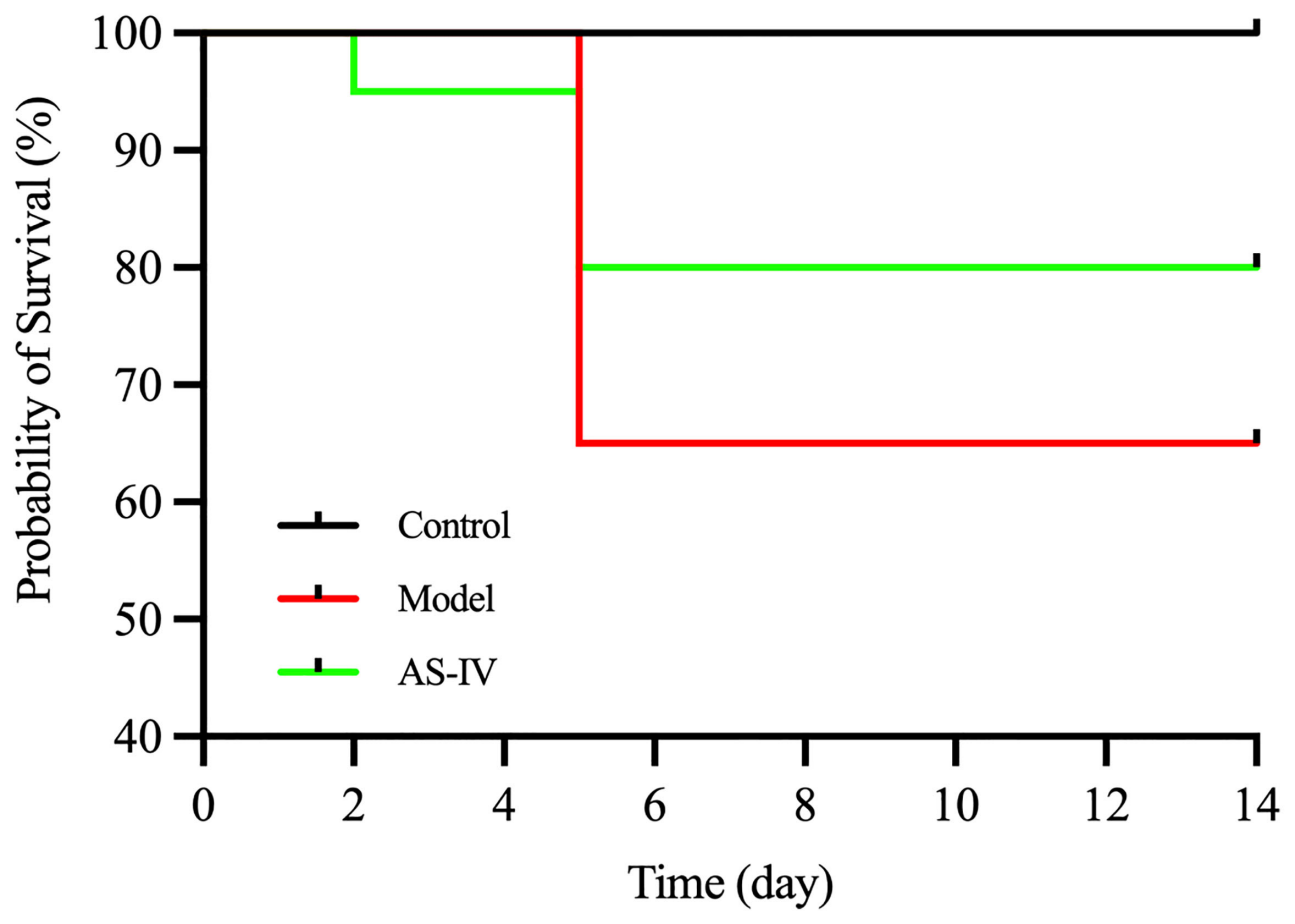
The survival rate in mice on day 14.

As shown (**Figure 2**), the survival rate was recorded at 65% for the model group on day 14, based on Kaplan-Meier survival curves. The survival rate for the AS-IV treatment group was 80% on day 14. In contrast, no mortality was observed in the control group.

### AS-IV Prevented ISO-Induced Cardiac Dysfunction

**Figure 3:**
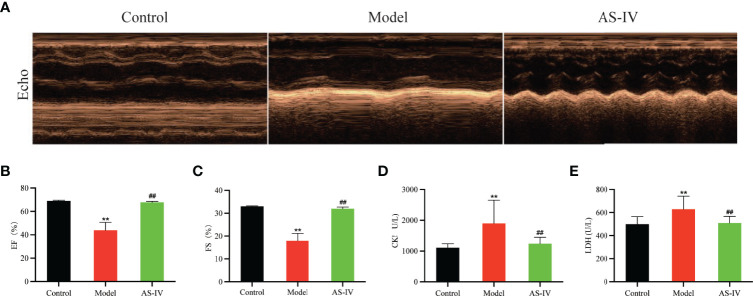
Effects of AS-IV on ISO-induced cardiac dysfunction and myocardial damage in mice. **(A)** Representative echocardiography; **(B, C)** Echocardiographic parameters, including EF and FS (n = 6); **(D, E)** Serum CK and LDH (n = 8). Mean ± SD. **P < 0.01 (the control vs. the model group). ^##^ *P* < 0.01 (the AS-IV vs. the model group).

Echocardiography was performed at the end of 14 days of AS-IV treatment. Echocardiography demonstrated that cardiac performance of the model group (*P* < 0.01) was significantly poor while EF and FS sharply were increased in the AS-IV group (*P* < 0.01), displaying enhancement of myocardial function (**Figures 3A–C**), an observation similar to Lu et al. (2018).

### AS-IV Attenuated ISO-Induced Myocardial Damage

The CK and LDH are markers of myocardial damage. The degree of myocardial damage is determined by measuring CK and LDH activities in mice serum. In **Figures 3D, E**, the levels of CK and LDH in the AS-IV group were markedly depressed compared with the Model group (*P*< 0.01), suggesting that AS-IV could facilitate improvements in myocardial damage induced by ISO. These results were consistent with the study reporting that AS-IV decreased CK and LDH levels in a cardiac fibrosis model (Lin et al., 2019).

### AS-IV Ameliorated ISO-Induced Histopathological Changes

**Figure 4:**
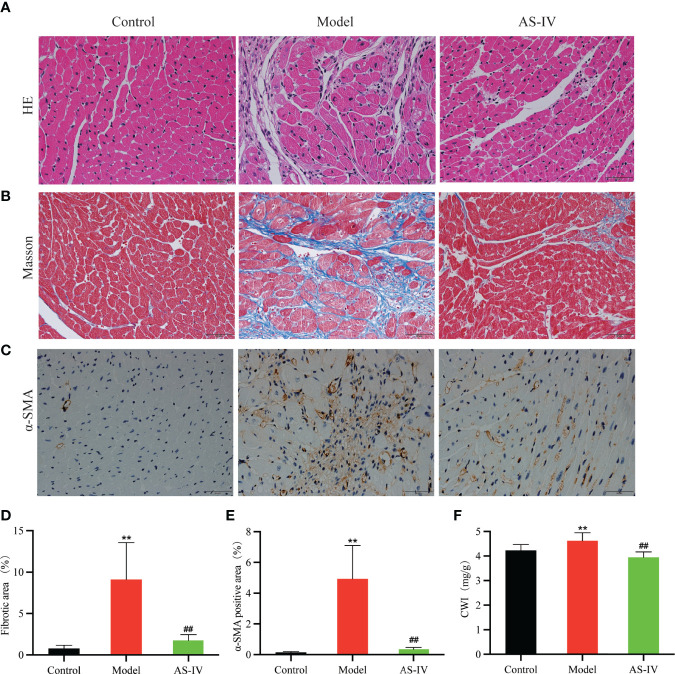
Effects of AS-IV on ISO-induced histopathological changes and cardiac fibrosis. **(A–C)** Representative images of cardiac fibrosis reflected by HE, Masson, and immunohistochemical staining. **(D, E)** Quantification of interstitial fibrosis and α-SMA (n = 8). **(F)** CWI. Mean ± SD. **P < 0.01(the control vs. the model group). ^##^ *P* < 0.01 (the AS-IV vs. the model group).

Histological analysis of heart tissues and HE stained cardiac sections of the Model group showed inflammatory cell infiltration, myocardial fiber thickening, myocardial necrosis, and myocardial structural disorder (**Figure 4A**), indicating myocardial damage. However, AS-IV administration caused an amelioration in this pathological change, displaying that AS-IV can protect against ISO-induced CF.

### AS-IV Alleviated ISO-Induced Cardiac Fibrosis

Cardiac fibrosis in ISO-treated mice was evident assessed histologically by collagen-specific Masson staining and chemically by expressions of α-SMA (Liu et al., 2019; Pan et al., 2019). As shown in **Figures 4B, D**, Masson staining analysis of cardiac tissue in the model group revealed significantly higher myocardial collagen deposition in comparison with the control group (*P* < 0.01). AS-IV caused a significant decrease in collagen precipitation (*P* < 0.01). In **Figures 4C, E**, immunohistochemistry results showed that α-SMA expression in the model group was remarkably increased while the expression of α-SMA in the AS-IV group was decreased (*P* < 0.01). Histological examination of cardiac tissue stained by Masson and immunohistochemistry showed that a mice model of ISO-induced myocardial fibrosis had been successfully established, an observation similar to Yang et al. (2020). As shown in **Figure 4F**, the CWI distinctly was increased in the group exposed to ISO (*P* < 0.01), while pretreatment with AS-IV reduced the CWI (*P* < 0.01).

### AS-IV Improved ISO-Induced Gut Microbial Dysbiosis

**Figure 5:**
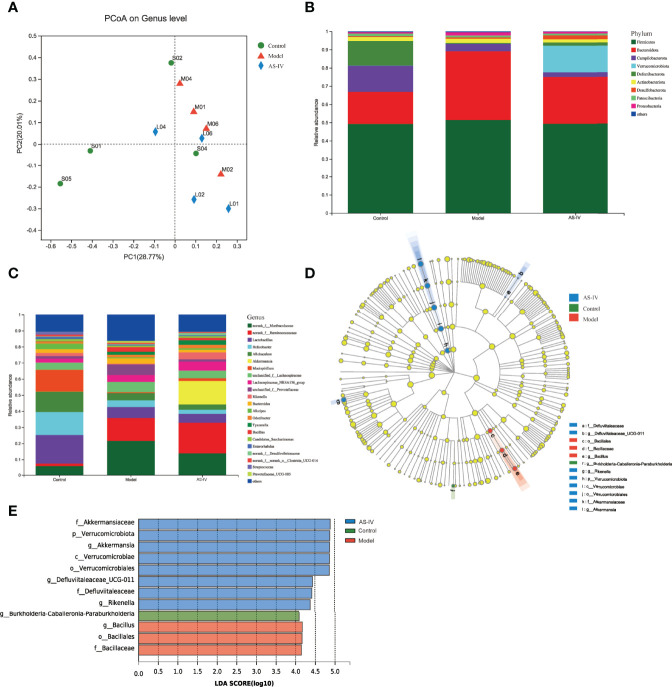
Effects of AS-IV on the structure of microbiota. **(A)** Principal coordinate analysis (PCoA). **(B, C)** Relative abundance of intestinal microbes at the phylum and genus level, respectively. **(D)** Taxonomy cladogram. **(E)** Gut microbial taxa (LDA threshold of 4.0).

The 16S rRNA sequencing analysis was conducted to determine whether AS-IV treatment affects intestinal microbial community composition. As the principal coordinate analysis (PCoA) shown, we could figure that microbial structures of the model group were clearly separated from those of the control and AS-IV groups (**Figure 5A**); the PC1 and PC2 explained 28.77% and 20.01% of the variation, respectively.

In **Figures 5B, C**, the fecal-microbe community distributions are shown. At the phylum level, the dominant microbial communities identified in this study in three groups were Bacteroidetes and Firmicutes (**Figure 5B**). Among these, the relative microbe abundances of Campilobacterota and Deferribacterota were reduced, and Bacteroidetes abundance of the model group was increased, but the abundance of Bacteroidetes was then reduced by AS-IV treatment (**Figure 5B**). Furthermore, the Verrucomicrobia abundance of the AS-IV group was increased relative to the model group (**Figure 5B**). At the genus level, Ruminococcaceae and Akkermansia abundances were the highest in the AS-IV group (**Figure 5C**). When compared to the model group, the relative abundance of Akkermansia at the genus level was increased by AS-IV treatment (**Figure 5C**).

Changes in intestinal microbial composition in different groups were also explored using the LEfSe analysis. As shown in the taxonomy cladogram and LDA scores (**Figures 5D, E**), the phylum Verrucomicrobiota, the genus Akkermansia, the genus Defluviitaleaceae_UCG-011, and the genus Rikenella were abundant in the fecal samples of the AS-IV group. These data show that AS-IV treatment influenced gut microbiota community structure, which could be beneficial to the host.

### AS-IV Improved ISO-Induced Fecal Metabolic Disorders

**Figure 6:**
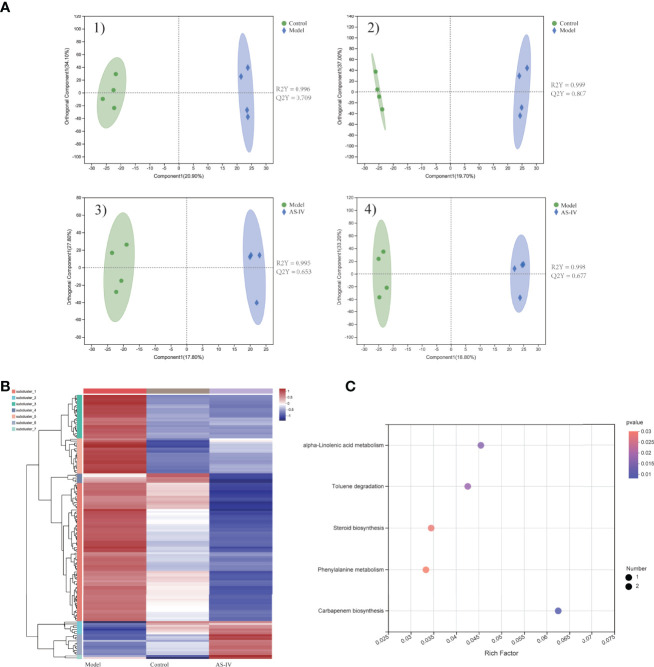
Metabolic profiles of feces samples in cardiac fibrosis mice treated by AS-IV. **(A)** 1,2) OPLS-DA plot of control and model groups in positive and negative mode; 3,4) OPLS-DA plot of model and AS-IV groups in positive and negative mode. **(B)** Heatmap of 141 significantly different metabolites. **(C)** Metabolic-pathway enrichment analysis.

The metabolic profiles of feces samples in three groups were analyzed by LC-MS. Separations between different groups were clearly shown in the OPLS-DA analysis (**Figure 6A**). When compared to the model group, 141 metabolites in the feces of the AS-IV treatment group were distinctly altered. Of 141 altered metabolites, 20 were upregulated while 121 were downregulated in the AS-IV group (**Table S1**). A heatmap was adopted for identifying different clusters of 141 significantly altered metabolites in three groups (**Figure 6B**). All 141 differentially expressed metabolites were subjected to pathway enrichment analysis. These metabolic markers were mainly involved in alpha-Linolenic acid metabolism, Toluene degradation, Steroid biosynthesis, Phenylalanine metabolism, and Carbapenem biosynthesis (**Figure 6C**).

### Correlation Between Specific Gut Microbes and Altered Fecal Metabolites

**Figure 7:**
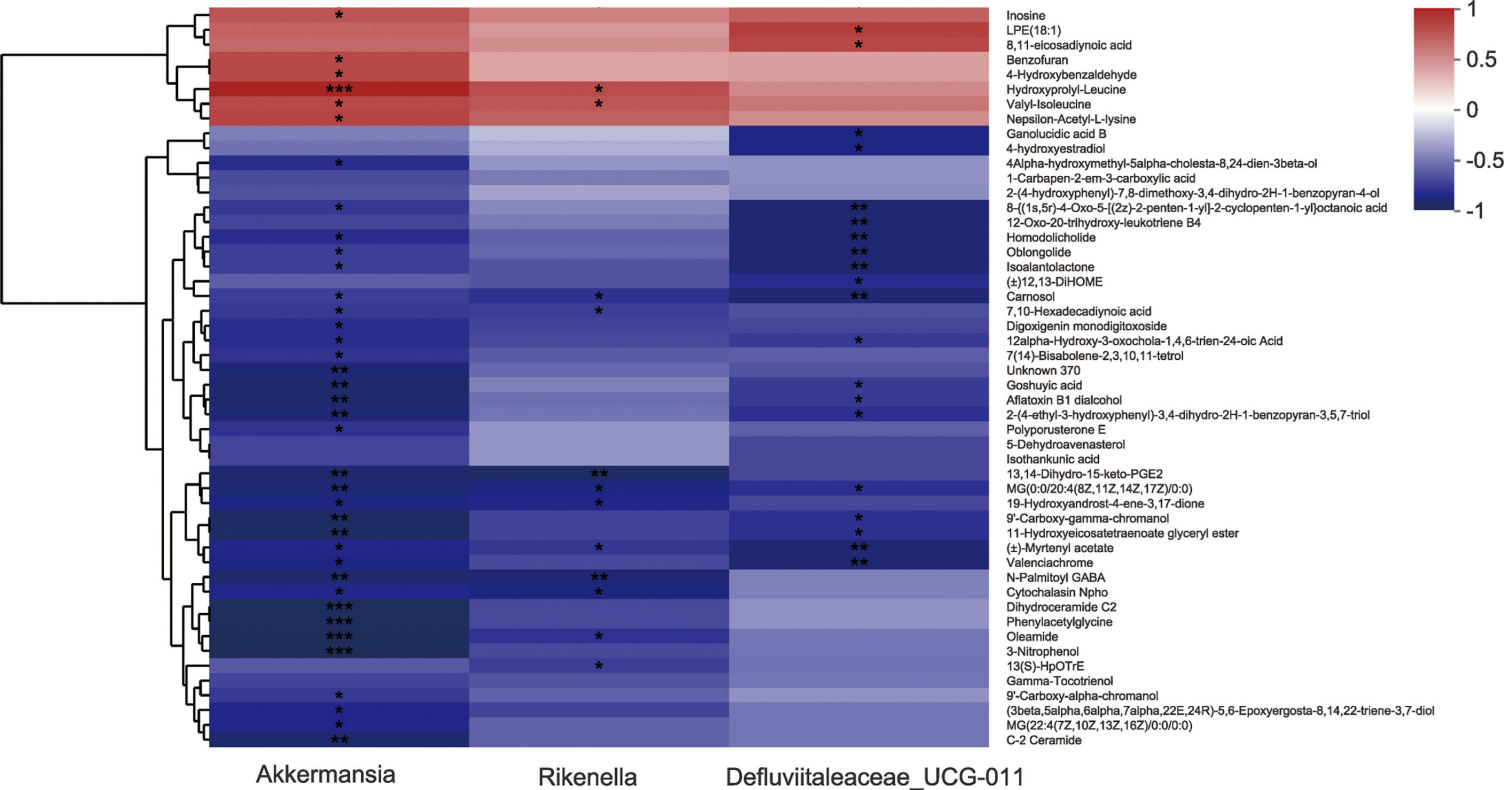
Correlation analysis between several specific intestinal microbes and altered fecal metabolites. *P<0.05, **P<0.01, and ***P<0.001 (the AS-IV vs. the model group).

16S rRNA sequencing data show that AS-IV consumption increased the gut Akkermansia, Defluviitaleaceae_UCG-011, and Rikenella in ISO-induced cardiac fibrosis mice. Metabolomics analysis data reveal that AS-IV administration ameliorated metabolic profiles in the stool. We further discussed the correlation between specific gut microbes and altered fecal metabolites through Spearman’s correlation analysis (**Figure 7**). Correlation analysis shows that specific intestinal microbes had high correlations with typical metabolic products. For example, gut Akkermansia had positive correlations with hydroxyprolyl-leucine, valyl-isoleucine, and nepsilon-acetyl-L-lysine, but negative correlations with phenylacetylglycine. These data suggest that gut-microbe composition specifically affected metabolic products in the stool.

## Discussions

Changes in gut microbiota are tightly linked to cardiovascular diseases, including cardiac fibrosis (Battson et al., 2018; Du et al., 2020). Compelling evidence has suggested that Astragaloside IV has anti-fibrosis and myocardial damage alleviating effects and aids in the treatment of cardiovascular diseases (Wan et al., 2018; Jiang et al., 2019; Tan et al., 2020). However, the role of AS-IV treatment in gut microbiota and microbial metabolites in ISO-induced CF mice has been unexplored. In this study, we explored the anti-fibrosis effects of AS-IV on ISO-induced mice and discussed the possibility of these effects mediated by intestinal microbiota and fecal metabolites. We demonstrated that treatment with AS-IV significantly alleviated ISO-induced cardiac dysfunction, cardiac fibrosis, and myocardial damage, demonstrated by the increasing of EF%, FS%, and the decreasing of CWI and serum CK, LDH. HE staining displayed that after AS-IV treatment, the myocardial structure tended to be normal, and the inflammatory cell infiltration and myocardial necrosis decreased. Masson staining showed that AS-IV markedly inhibited ISO-triggered collagen deposition. Moreover, immunohistochemistry results proved that α-SMA expression related to collagen synthesis in cardiac tissues was also decreased after AS-IV treatment. A previous report by Wan et al. (2018) demonstrated that AS-IV ameliorated ISO-triggered CF in mice, which is consistent with our data.

Gut microbiota is presumed to contribute much to regulating host metabolic pathways (Fischbach and Segre, 2016) and is of crucial importance to cardiac fibrosis (Du et al., 2020). In this study, Bacteroidetes abundance was increased in CF model group mice relative to the control group mice, but the abundance of Bacteroidetes was reduced after AS-IV treatment. This result agreed with the alterations of gut microbiota in the treatment of ventricular remodeling with Chinese herbal medicine (Yang et al., 2019). Strikingly, we found that Akkermansia, Defluviitaleaceae_UCG-011, and Rikenella abundances were markedly increased in ISO-induced CF mice treated with AS-IV, which is consistent with the findings of a published study where drug-administered increased the genus Defluviitaleaceae_UCG-011 in hyperlipidemic rats (Zhang et al., 2021), and in contrast to the outcomes of previous studies where drug-administered decreased the genus Rikenella in high-fat diet mice (He W. S. et al., 2020; Liu J. et al., 2021). Akkermansia is the major species of A. muciniphila and represented the cultivated gut phylum Verrucomicrobia (Derrien et al., 2017). Akkermansia is a beneficial gut commensal, which colonizes the gastrointestinal tract mucus layer, representing 1 to 4% of stool microbes (Macchione et al., 2019; Huck et al., 2020). It provides crucial host immunological responses, and its anti-inflammatory properties have been demonstrated (Macchione et al., 2019; Huck et al., 2020). Akkermansia can therefore be considered as a biomarker of intestinal environmental health (Belzer and de Vos, 2012). Emerging evidence also suggests that Akkermansia is associated with metabolic disorders and cardiovascular diseases (Lyu et al., 2017; Zhu et al., 2018). Akkermansia abundance is inversely associated with metabolic syndrome and atherogenesis in humans and mice (Li et al., 2016; Depommier et al., 2019). Moreover, Gutiérrez-Calabrés et al. reported that the abundance of Akkermansia was found diminished in spontaneously hypertensive heart failure model rats relative to both normotensive and spontaneously hypertensive rats (Gutiérrez-Calabrés et al., 2020). Considering these previous findings and our results, we regard that Akkermansia, Defluviitaleaceae_UCG-011, and Rikenella contribute to the anti-fibrosis effect of AS-IV in ISO-induced CF mice.

**Figure 8:**
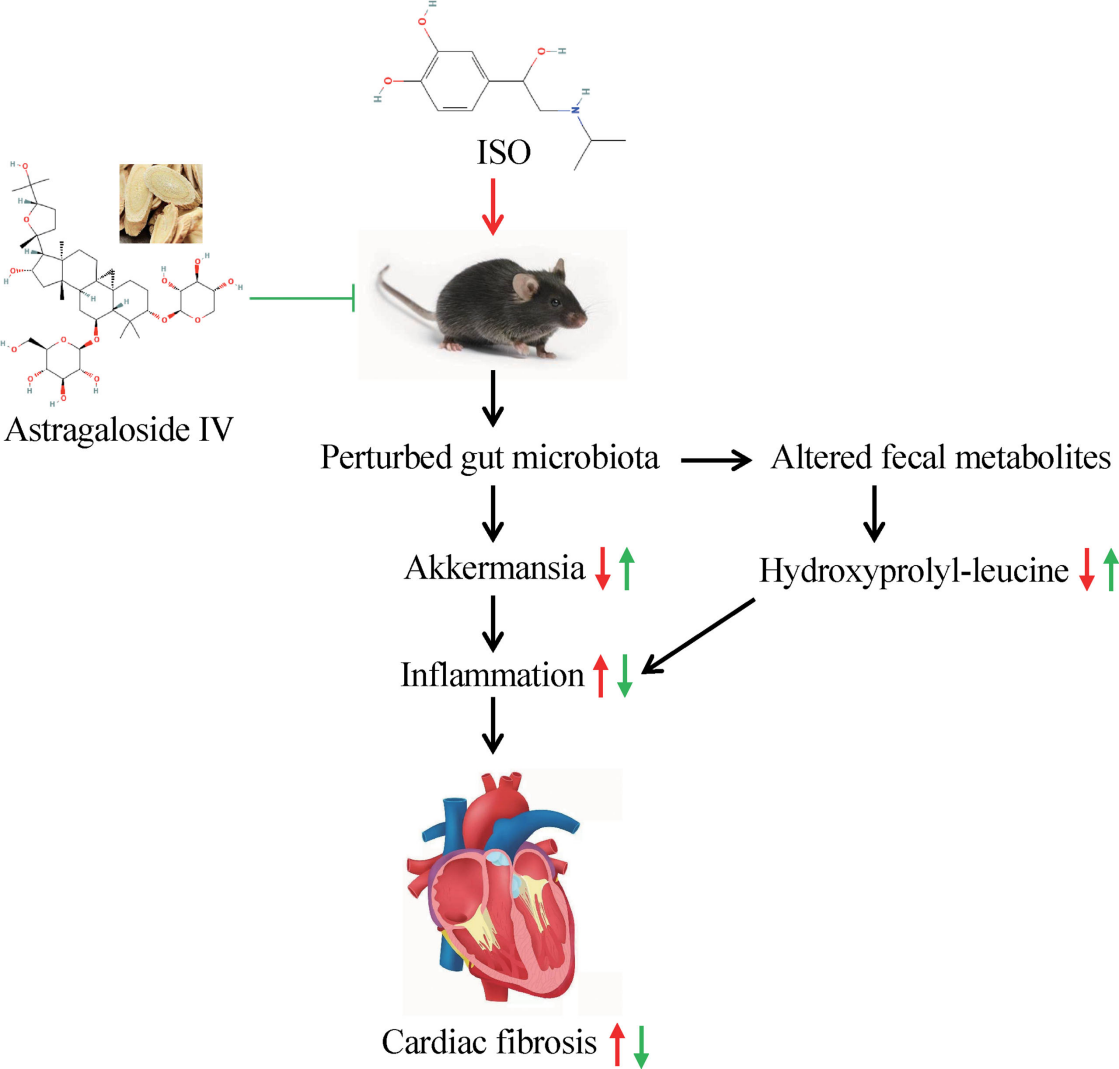
Mechanism of AS-IV on gut microbiota in ISO-induced cardiac fibrosis mice.

Significant alterations in the fecal metabolites were characterized after a 14-day AS-IV intervention. An important finding of fecal metabolomics was that the phenylalanine metabolism pathway was significantly enriched in AS-IV-treated mice relative to the model group mice. Phenylalanine catabolism disorder, resulting in elevated plasma levels of the indispensable amino acid phenylalanine, was positively associated with macrovascular disease risk (Würtz et al., 2015; Czibik et al., 2021), and the levels of plasma phenylalanine were observed to increase in HF patients (Chen et al., 2020; Cheng et al., 2021). In addition, previous reports by Liu X. et al. (2021) demonstrated that Akkermansia decreased phenylalanine levels while increasing leucine and valine levels. We also observed correlative changes of several specific gut microbes with the abundance of altered fecal metabolites. For example, Akkermansia had positive correlations with hydroxyprolyl-leucine, valyl-isoleucine, and nepsilon-acetyl-L-lysine, but negative correlations with phenylacetylglycine. These results indicate that AS-IV has anti-fibrosis effects in ISO-induced cardiac fibrosis mice and that these effects may be mediated by alterations in intestinal microbiota and fecal metabolites (**Figure 8**).

## Conclusion

Collectively, our study adds to the accumulating evidence that Astragaloside IV ameliorates cardiac fibrosis and the impaired heart function induced by ISO. Our findings support the hypothesis that alterations in intestinal microbiota and fecal metabolites contribute to the anti-fibrosis activity of Astragaloside IV. Our study first reveals that Astragaloside IV sharply increased the abundance of Akkermansia, Defluviitaleaceae_UCG-011, and Rikenella, thereby affecting phenylalanine metabolism to achieve curative effects on cardiac fibrosis mice. Our data raises strong evidence for the application of Astragaloside IV in the treatment of myocardial fibrosis.

## Data Availability Statement

The datasets presented in this study can be found in online repositories. The names of the repository/repositories and accession number(s) can be found below: NCBI SRA database, accession number PRJNA811352, and MetaboLights database, accession number MTBLS4359.

## Ethics Statement

The animal study was reviewed and approved by the Animal Experiments Ethical Review Committee of Chongqing Medical University (Chongqing, China) under No. 2021040.

## Author Contributions

W-FC and X-QD designed the experiments. X-QD and L-PS carried out most of the experiments. J-YH, BZ, and YX performed parts of the experiments. X-QD and L-PS collected and analyzed data. X-QD contributed to the writing of the original draft. Z-WC and L-PS provided guidance for software and figures. All authors reviewed and approved the final article.

## Funding

This work was supported by Chongqing Medical University Postdoctoral Foundation (No. R11004), Chongqing Postdoctoral Science Foundation (No. cstc2021jcyj-bshX0215), and Xinglin program of Chongqing TCM/TCM-integrated Key discipline (No. 2021-ZDXK-bshx03, No.2021-ZDXK-DB03).

## Conflict of Interest

The authors declare that the research was conducted in the absence of any commercial or financial relationships that could be construed as a potential conflict of interest.

## Publisher’s Note

All claims expressed in this article are solely those of the authors and do not necessarily represent those of their affiliated organizations, or those of the publisher, the editors and the reviewers. Any product that may be evaluated in this article, or claim that may be made by its manufacturer, is not guaranteed or endorsed by the publisher.
