# Supplementary figures and images for: Astragaloside IV Ameliorates Isoprenaline-Induced Cardiac Fibrosis in Mice via Modulating Gut Microbiota and Fecal Metabolites
Source: Front Cell Infect Microbiol. 2022 May 17;12:836150. doi: 10.3389/fcimb.2022.836150 (PMC9152365; doi:10.3389/fcimb.2022.836150)

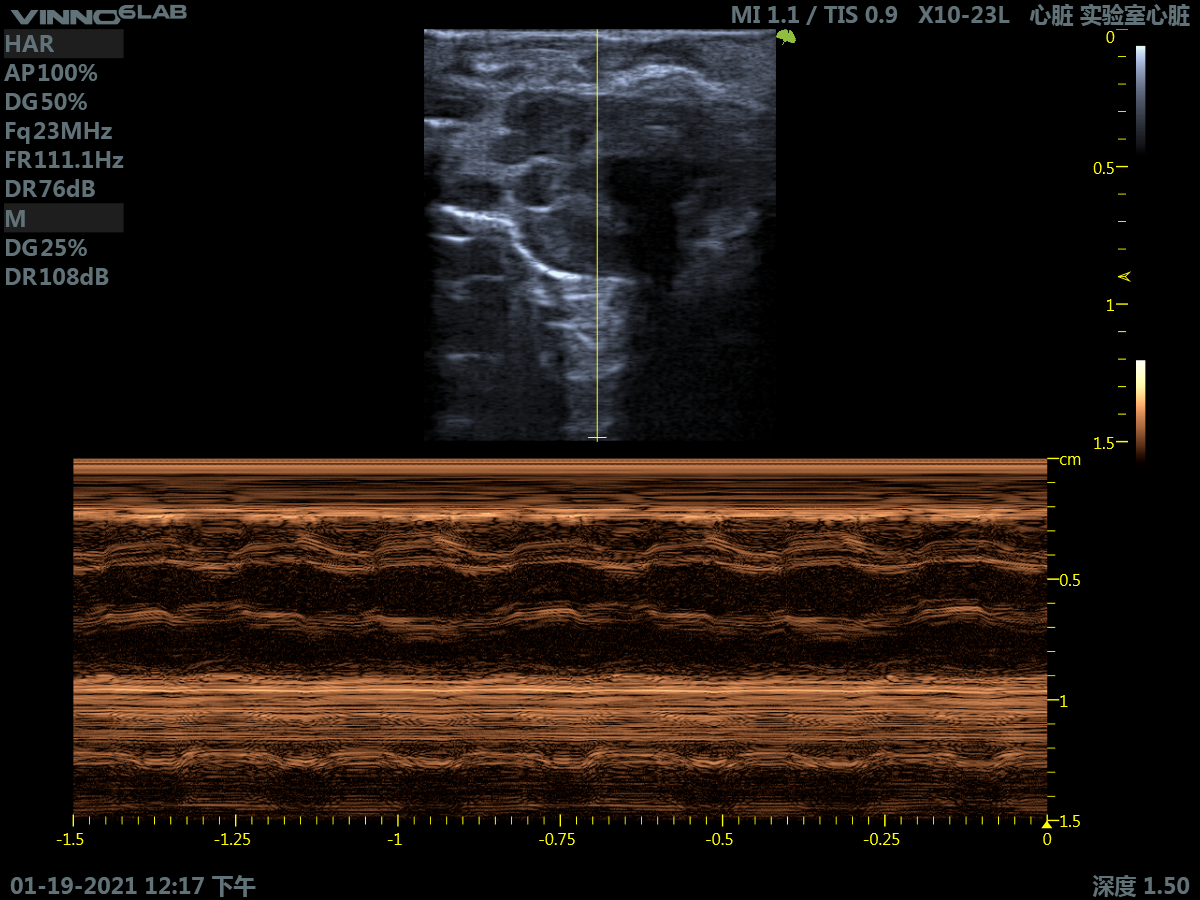

Supplement: Supplementary file 1 [file Image_1.tif]

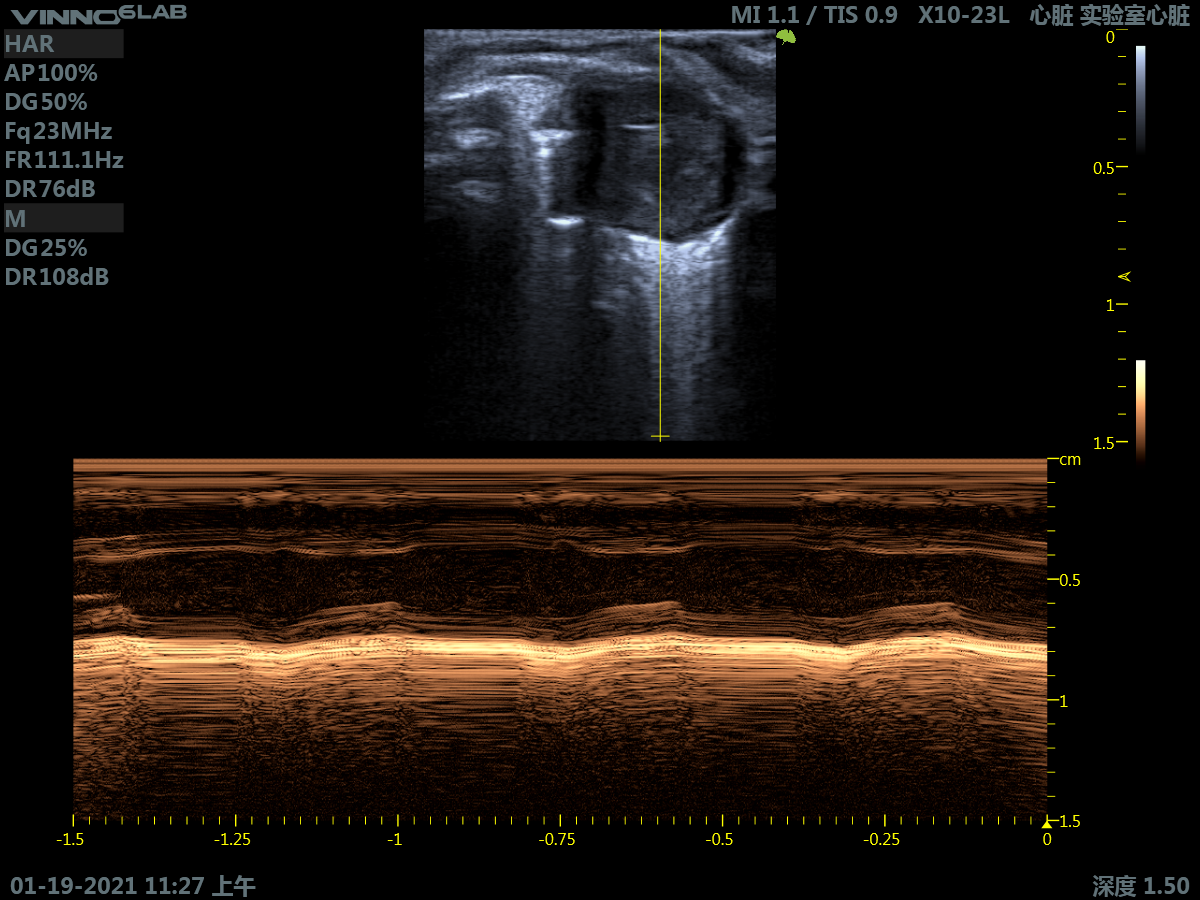

Supplement: Supplementary file 2 [file Image_2.tif]

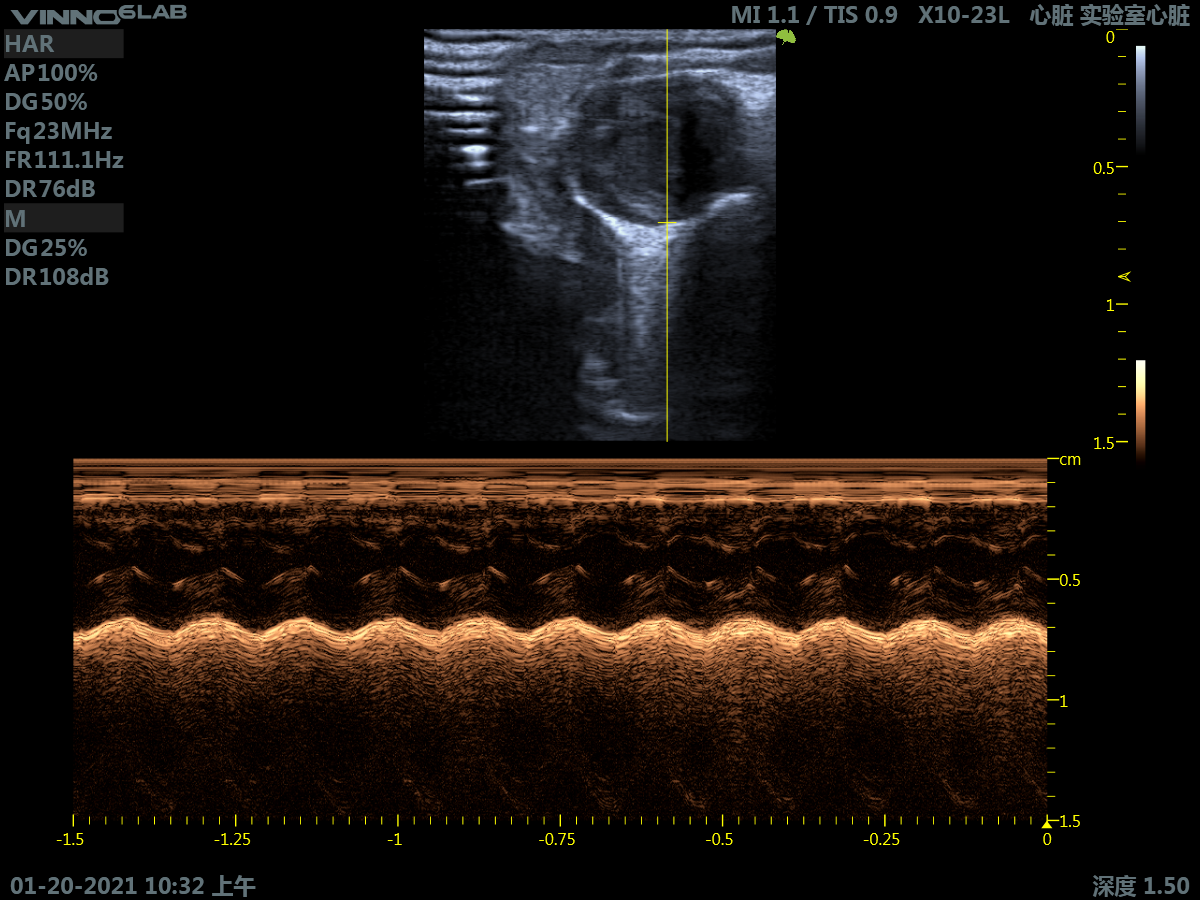

Supplement: Supplementary file 3 [file Image_3.tif]

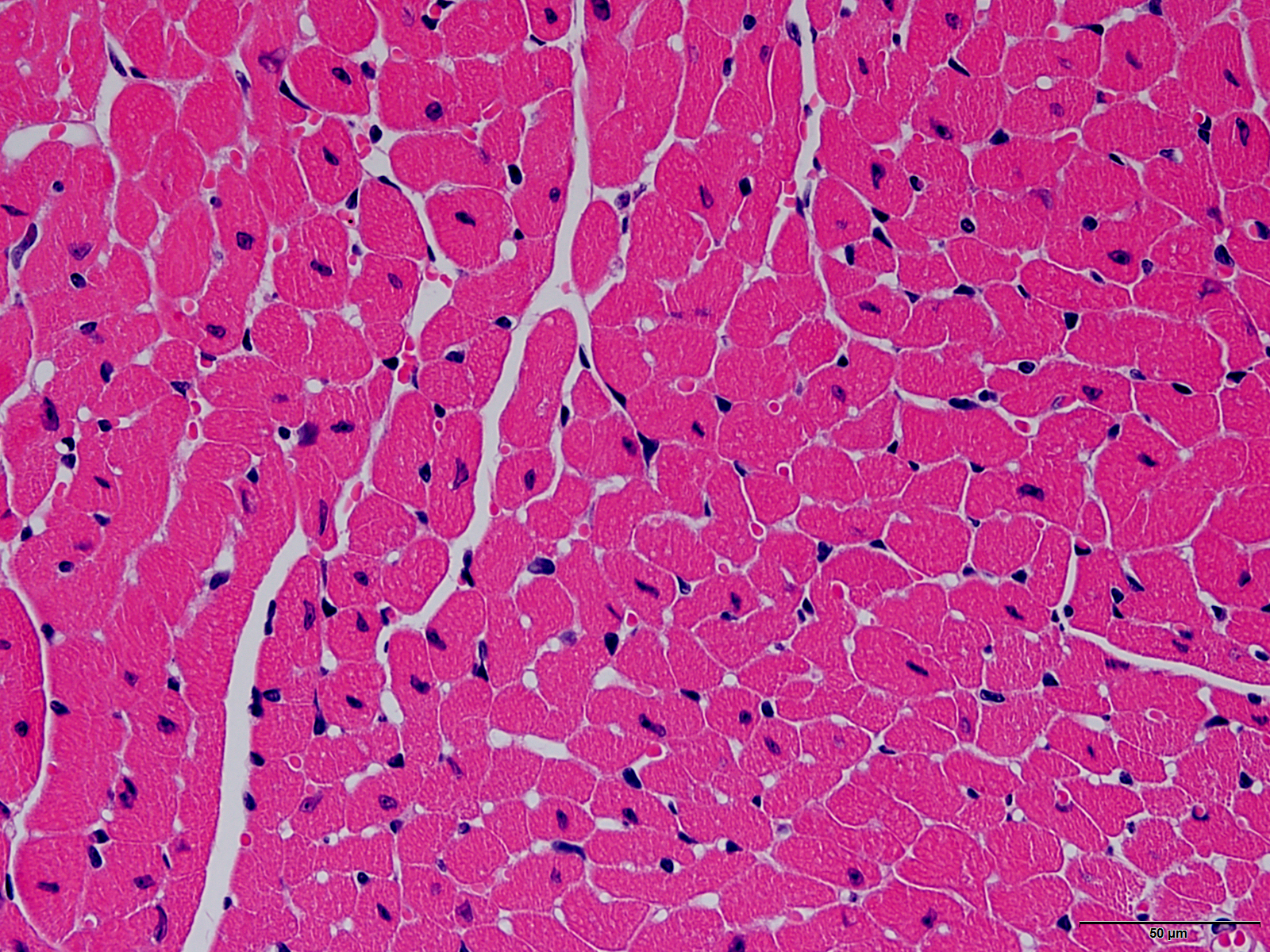

Supplement: Supplementary file 4 [file Image_4.tif]

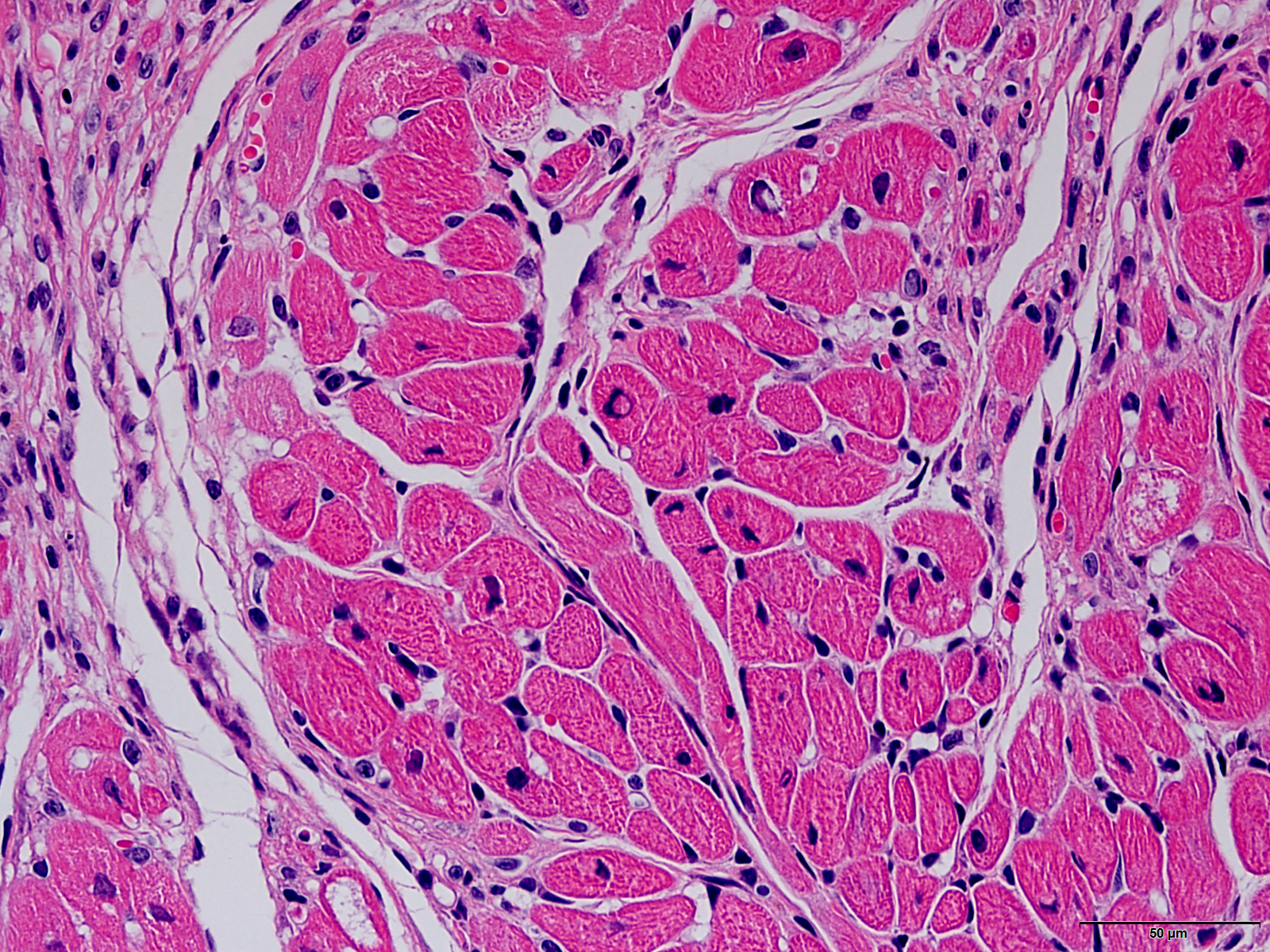

Supplement: Supplementary file 5 [file Image_5.tif]

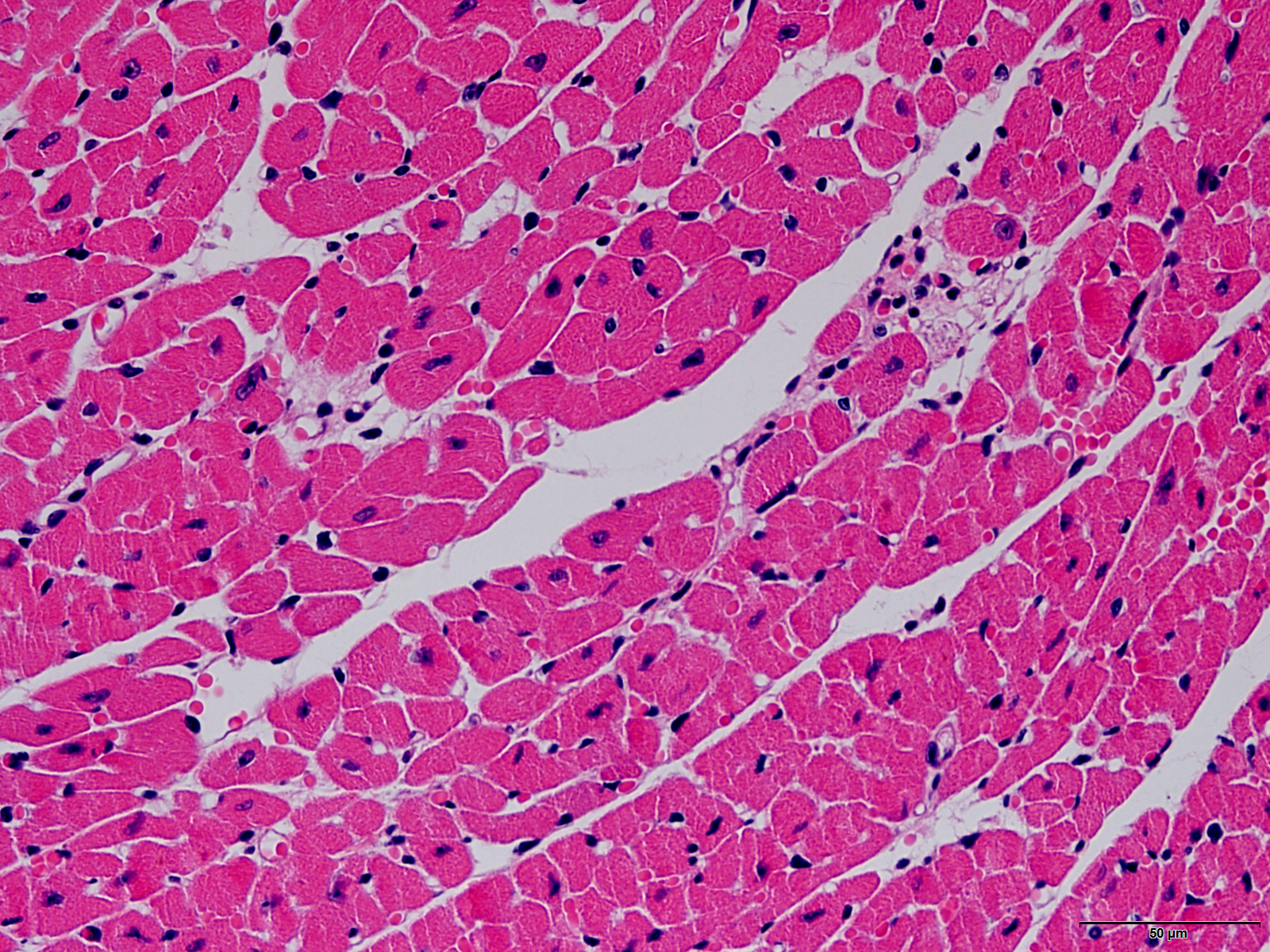

Supplement: Supplementary file 6 [file Image_6.tif]

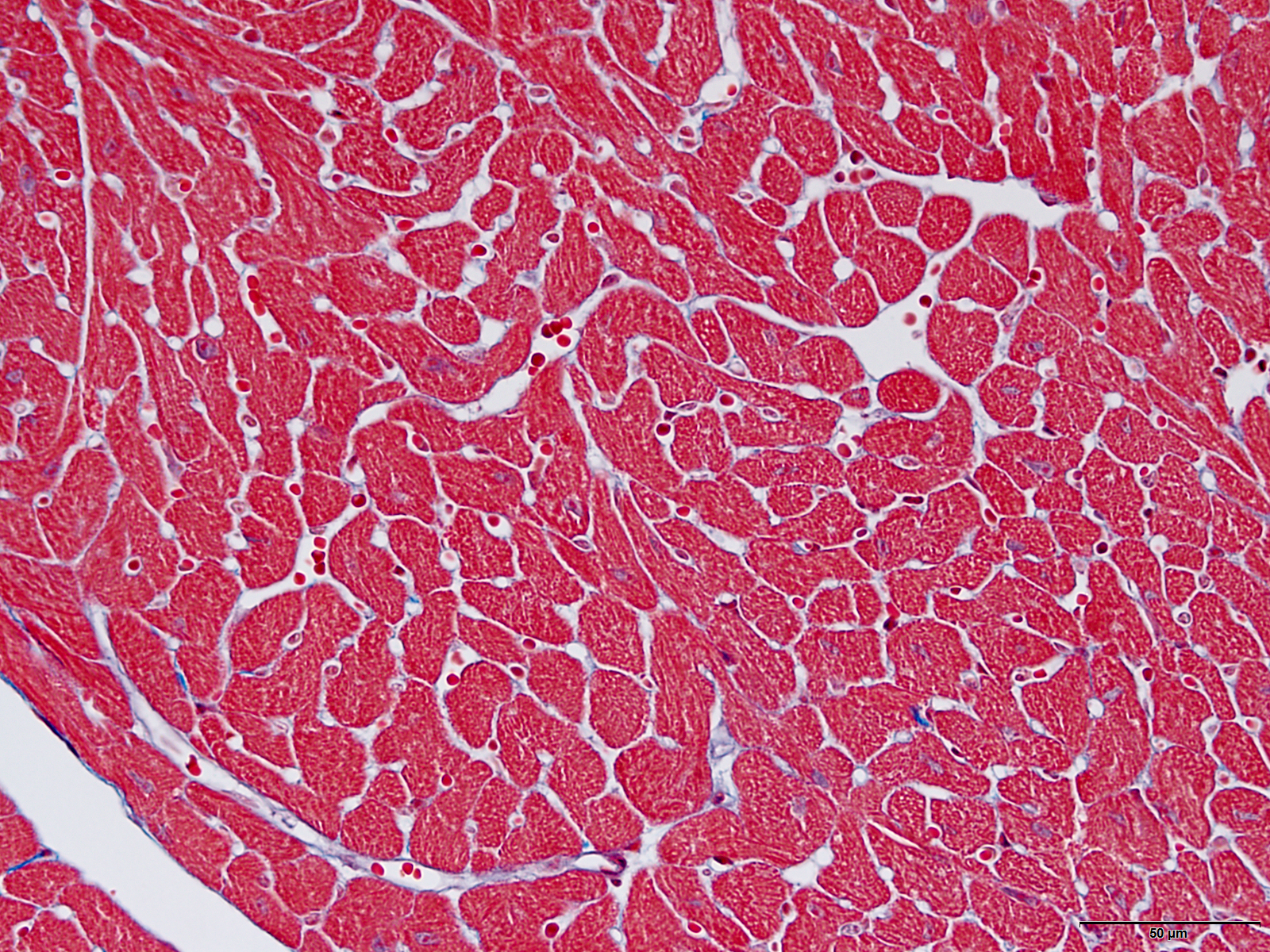

Supplement: Supplementary file 7 [file Image_7.tif]

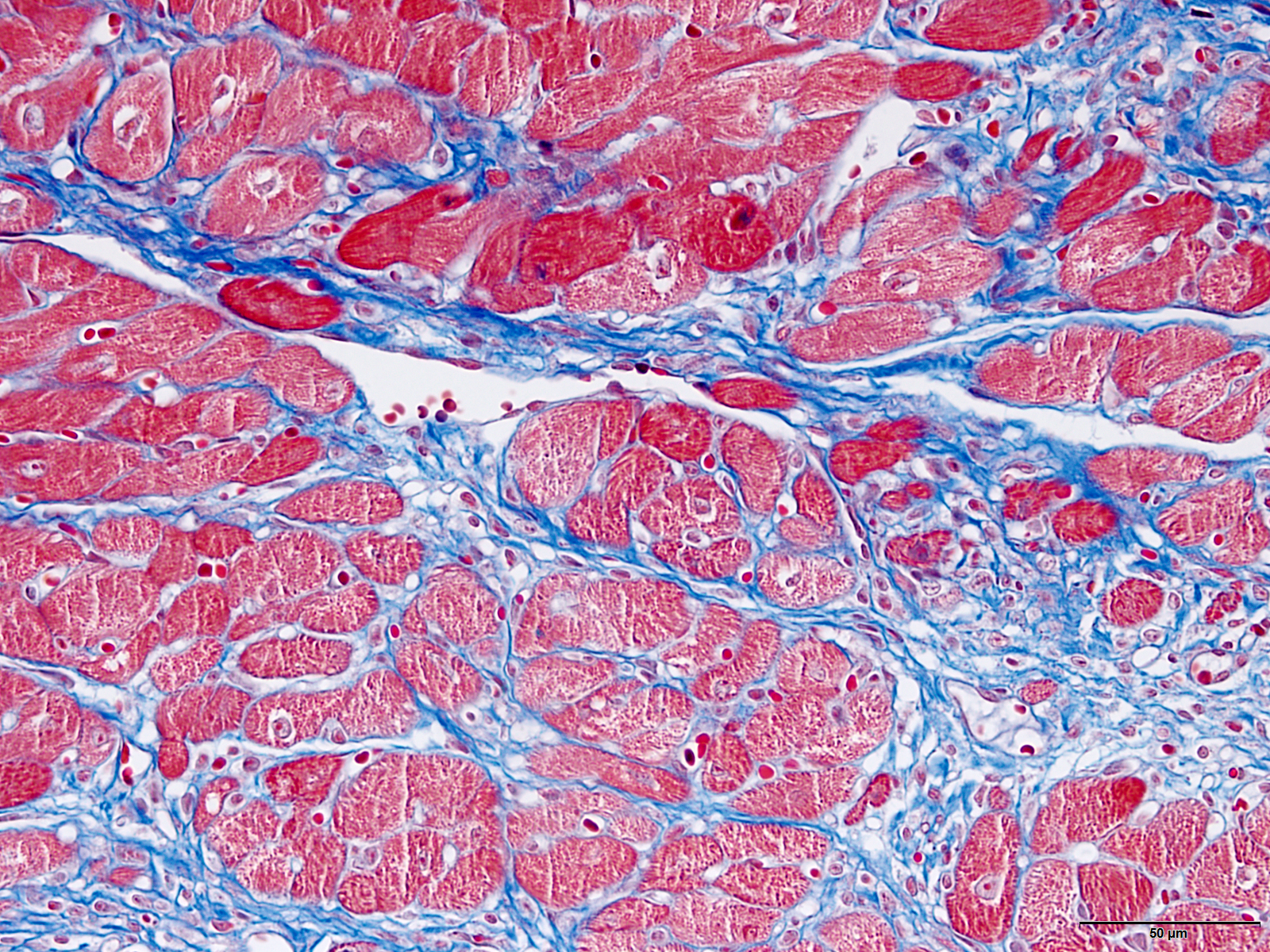

Supplement: Supplementary file 8 [file Image_8.tif]

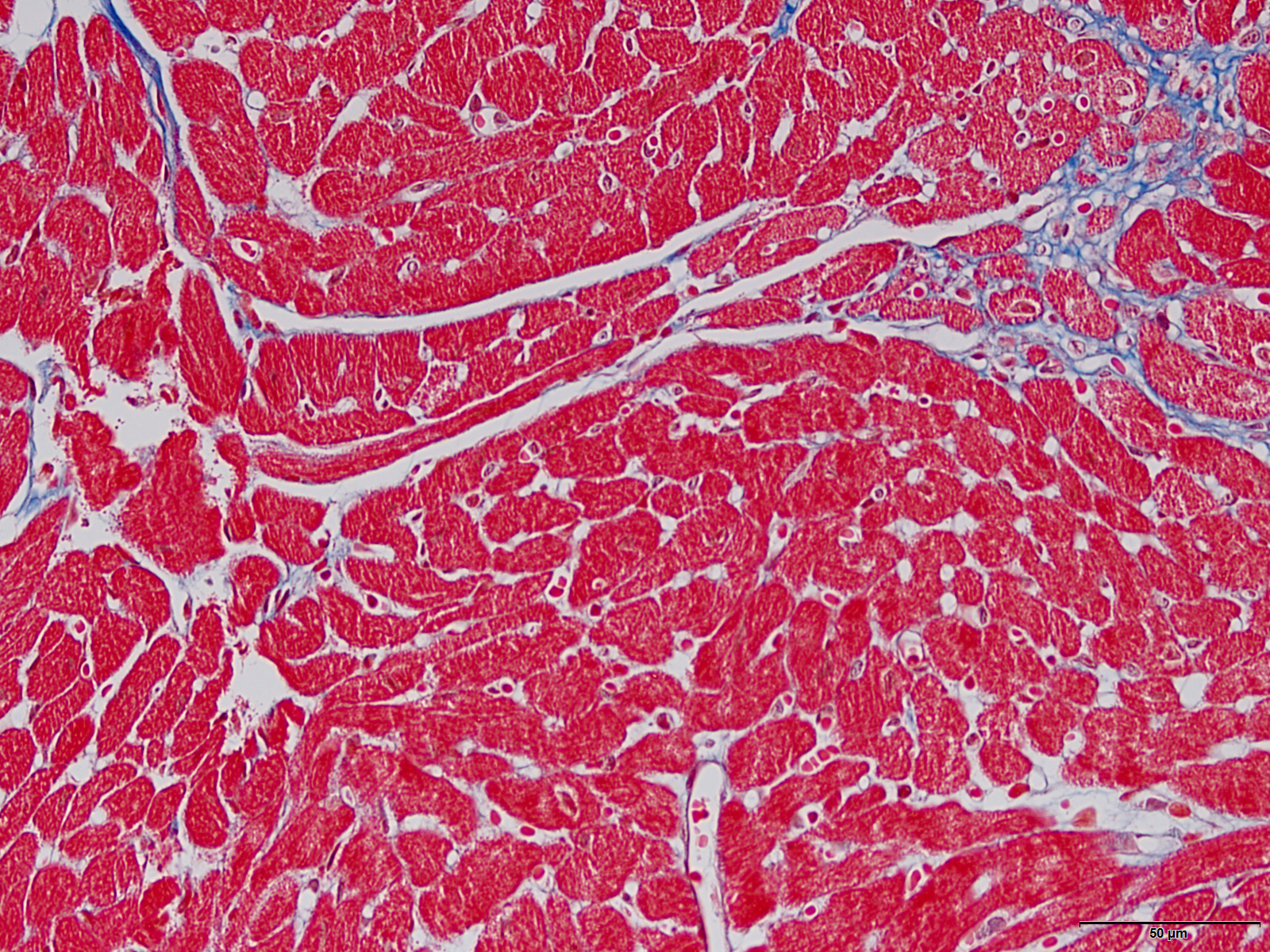

Supplement: Supplementary file 9 [file Image_9.tif]

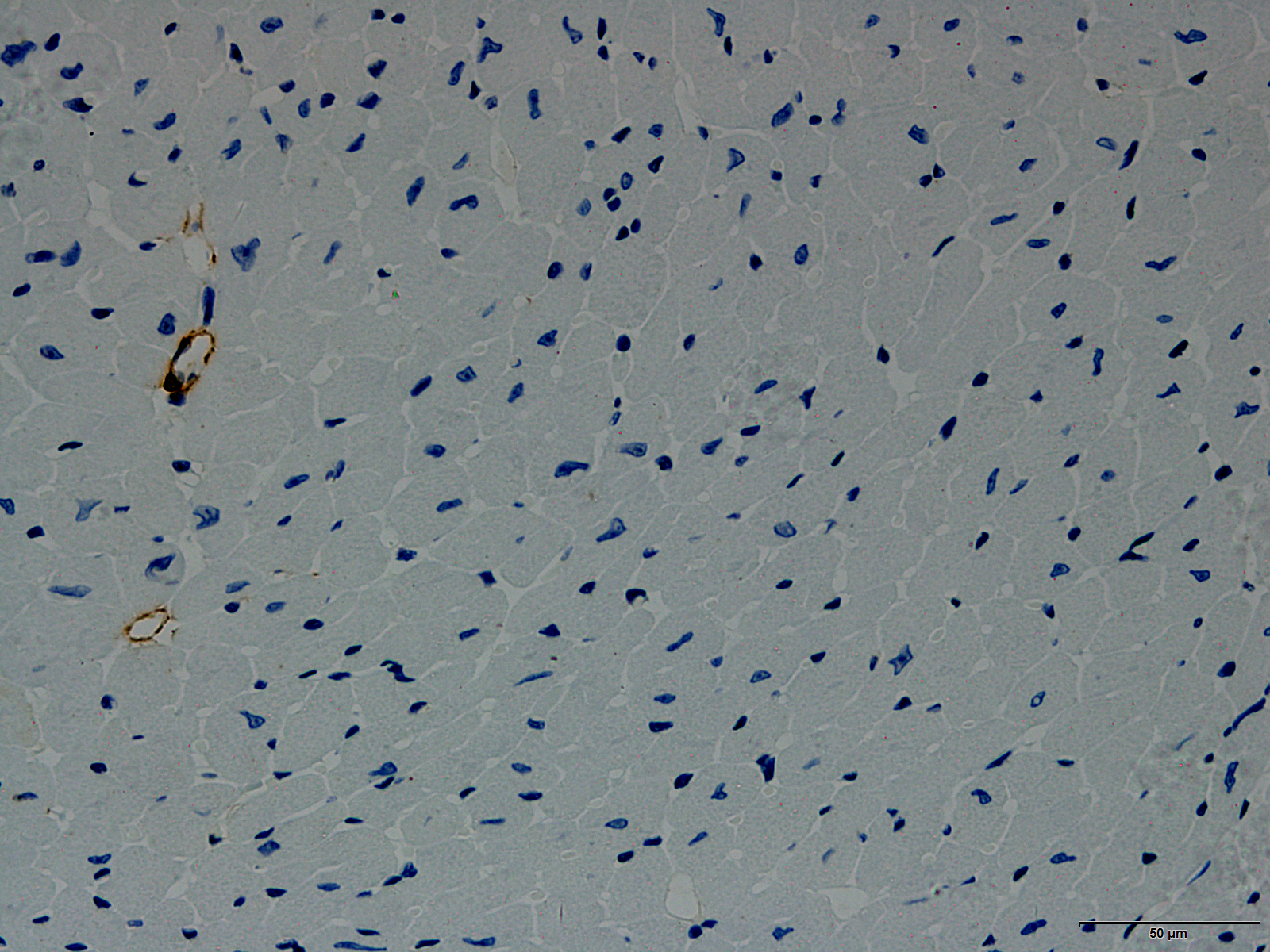

Supplement: Supplementary file 10 [file Image_10.tif]

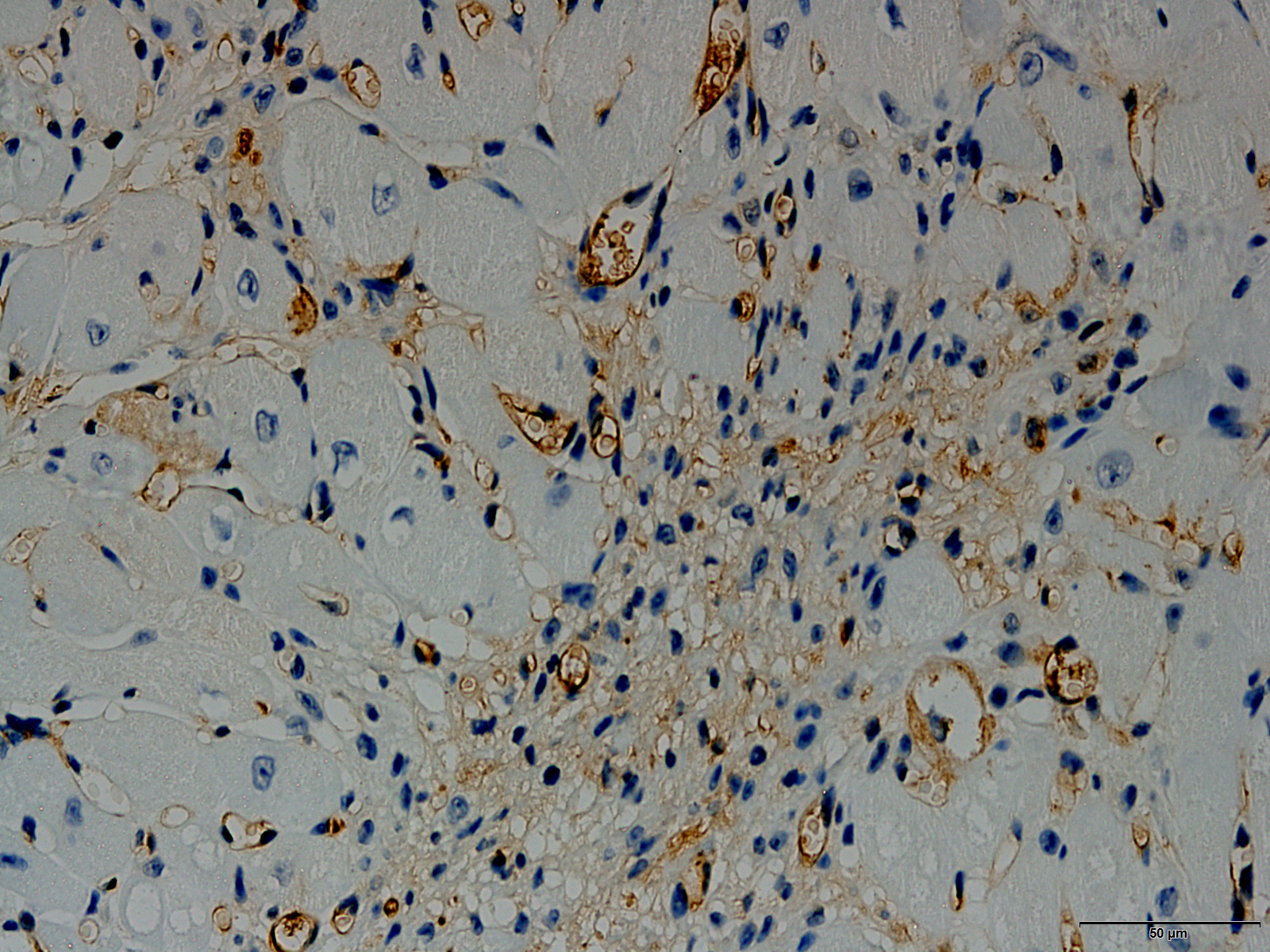

Supplement: Supplementary file 11 [file Image_11.tif]

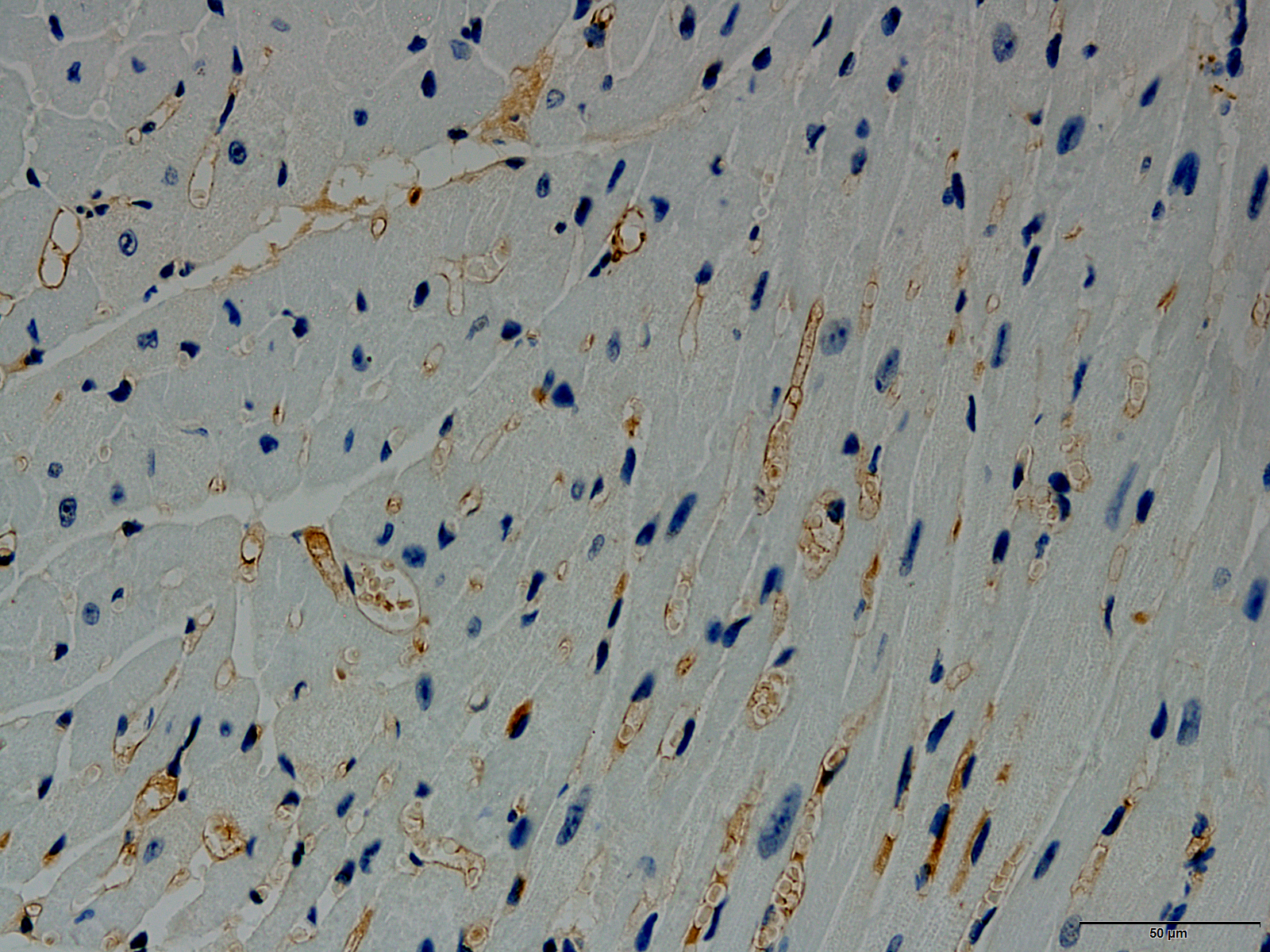

Supplement: Supplementary file 12 [file Image_12.tif]
