# Supplementary material for: Astragaloside IV Ameliorates Isoprenaline-Induced Cardiac Fibrosis in Mice via Modulating Gut Microbiota and Fecal Metabolites
Source: Front Cell Infect Microbiol. 2022 May 17;12:836150. doi: 10.3389/fcimb.2022.836150 (PMC9152365; doi:10.3389/fcimb.2022.836150)
Supplement: Supplementary file 13 [file Table_1.docx]

**Table S1 |** 141 significantly altered metabolites.

| Metabolite | Metab ID | FC(AS-IV/Model) | P-value | up/down |
| --- | --- | --- | --- | --- |
| Taurochenodeoxycholate-7-sulfate | metab_19885 | 1.87373068 | 0.000903 | up |
| Pantetheine | metab_21289 | 1.46582499 | 0.03293 | up |
| Vestitone 7-glucoside | metab_3498 | 1.36702899 | 0.000198 | up |
| 1-(2,3-Dihydro-1H-pyrrolizin-5-yl)-1,4-pentanedione | metab_21513 | 1.34077156 | 0.01385 | up |
| 20-Hydroxy-leukotriene E4 | metab_22516 | 1.29148936 | 0.000464 | up |
| Rimexolone | metab_14689 | 1.2492388 | 0.02719 | up |
| Inosine | metab_22689 | 1.1728763 | 0.03148 | up |
| N-Acetyl-D-phenylalanine | metab_14657 | 1.152 | 0.03688 | up |
| Taurocholic acid 3-sulfate | metab_12494 | 1.13588324 | 0.03592 | up |
| Gamma-Glutamylproline | metab_13776 | 1.12845745 | 0.0055 | up |
| (E)-5-(3,4,5,6-Tetrahydro-3-pyridylidenemethyl)-2-furanmethanol | metab_14249 | 1.12565998 | 0.00447 | up |
| Cis-Uvariamicin IB | metab_16765 | 1.11068011 | 0.03888 | up |
| Hydroxyprolyl-Leucine | metab_11076 | 1.08628461 | 0.01593 | up |
| P-cresol | metab_927 | 1.07087899 | 0.04993 | up |
| 4-Hydroxybenzaldehyde | metab_11087 | 1.06856924 | 0.04564 | up |
| Benzofuran | metab_929 | 1.06482982 | 0.04521 | up |
| Nepsilon-Acetyl-L-lysine | metab_22413 | 1.0636928 | 0.04978 | up |
| LPE(18:1) | metab_18811 | 1.06354515 | 0.002164 | up |
| Valyl-Isoleucine | metab_22148 | 1.05771812 | 0.04825 | up |
| 8,11-eicosadiynoic acid | metab_18450 | 1.05442443 | 0.01096 | up |
| 4-hydroxyestradiol | metab_4059 | 0.96983006 | 0.03232 | down |
| Oblongolide | metab_8563 | 0.96851746 | 0.0205 | down |
| 7,10-Hexadecadiynoic acid | metab_4483 | 0.96741032 | 0.0179 | down |
| MG(22:4(7Z,10Z,13Z,16Z)/0:0/0:0) | metab_16336 | 0.96736083 | 0.0152 | down |
| Gibberellin A9 | metab_1355 | 0.96528085 | 0.02084 | down |
| Homodolicholide | metab_782 | 0.96503497 | 0.009872 | down |
| C14:5n-1,3,5,7,9 | metab_4803 | 0.9646955 | 0.04548 | down |
| 2,6Z-Nonadien-4-olide | metab_8875 | 0.96447876 | 0.01129 | down |
| All-trans-Farnesyl acetate | metab_1834 | 0.9633984 | 0.01802 | down |
| 13(S)-HpOTrE | metab_449 | 0.96332914 | 0.01065 | down |
| 8-{(1s,5r)-4-Oxo-5-[(2z)-2-penten-1-yl]-2-cyclopenten-1-yl}octanoic acid | metab_8578 | 0.96325817 | 0.009601 | down |
| ()-Myrtenyl acetate | metab_1945 | 0.96143864 | 0.01632 | down |
| Ingenol | metab_9298 | 0.96070368 | 0.003341 | down |
| 12-Oxo-20-trihydroxy-leukotriene B4 | metab_20853 | 0.96032072 | 0.006375 | down |
| 2-(4-hydroxyphenyl)-7,8-dimethoxy-3,4-dihydro-2H-1-benzopyran-4-ol | metab_22166 | 0.95905681 | 0.04321 | down |
| Indole-3-carboxaldehyde | metab_4836 | 0.95834385 | 0.01525 | down |
| Ganolucidic acid B | metab_2000 | 0.9583179 | 0.04693 | down |
| (3S,7E,9S)-9-Hydroxy-4,7-megastigmadien-3-one 9-glucoside | metab_9620 | 0.95805963 | 0.03567 | down |
| 1,2-Di(2-pyridyl)ethylene | metab_3618 | 0.95535248 | 0.02093 | down |
| Ent-17-Oxo-15-kauren-19-oic acid | metab_8992 | 0.95514394 | 0.01924 | down |
| Hydroxyibuprofen | metab_4382 | 0.95467137 | 0.02307 | down |
| Hexyl glucoside | metab_10328 | 0.95380645 | 0.01425 | down |
| LEVULINIC ACID, 3-BENZYLIDENYL- | metab_1515 | 0.95369746 | 0.006437 | down |
| 12-Oxo-2,3-dinor-10,15-phytodienoic acid | metab_8792 | 0.95206455 | 0.01286 | down |
| 5-Dehydroavenasterol | metab_5562 | 0.951255 | 0.04158 | down |
| 13,14-Dihydro-15-keto-PGE2 | metab_15805 | 0.95023844 | 0.01646 | down |
| (2'E,4'Z,7'Z,8E)-Colnelenic acid | metab_10498 | 0.95006688 | 0.01373 | down |
| Floribundoside | metab_12158 | 0.94924554 | 0.02017 | down |
| 5-(14-Nonadecenyl)-1,3-benzenediol | metab_7151 | 0.94878215 | 0.01363 | down |
| Phenylacetylglycine | metab_9410 | 0.94772089 | 0.04443 | down |
| 4,8 dimethylnonanoyl carnitine | metab_9929 | 0.94710095 | 0.01632 | down |
| 19-Hydroxyandrost-4-ene-3,17-dione | metab_20018 | 0.94669685 | 0.01164 | down |
| L-4-Hydroxy-3-methoxy-a-methylphenylalanine | metab_3621 | 0.94552529 | 0.03409 | down |
| 8-Deoxy-11,13-dihydroxygrosheimin | metab_15168 | 0.94442995 | 0.00657 | down |
| Carnosol | metab_4576 | 0.94283398 | 0.01802 | down |
| 7-beta-D-Glucopyranosyloxybutylidenephthalide | metab_14306 | 0.94214268 | 0.03091 | down |
| Pterosin N | metab_10565 | 0.94182894 | 0.02291 | down |
| Valenciachrome | metab_4426 | 0.94127344 | 0.01398 | down |
| Isoalantolactone | metab_8562 | 0.94030782 | 0.01948 | down |
| Austdiol | metab_21752 | 0.93790673 | 0.03557 | down |
| Aflatoxin B1 dialcohol | metab_1545 | 0.93781383 | 0.03572 | down |
| MG(22:6(4Z,7Z,10Z,13Z,16Z,19Z)/0:0/0:0) | metab_16142 | 0.93735849 | 0.007306 | down |
| ()12,13-DiHOME | metab_2176 | 0.93684005 | 0.001092 | down |
| 4Alpha-hydroxymethyl-5alpha-cholesta-8,24-dien-3beta-ol | metab_18668 | 0.93541396 | 0.02486 | down |
| MG(0:0/20:4(8Z,11Z,14Z,17Z)/0:0) | metab_20123 | 0.93536265 | 0.0296 | down |
| 9'-Carboxy-gamma-chromanol | metab_20297 | 0.93486786 | 0.02374 | down |
| 7(14)-Bisabolene-2,3,10,11-tetrol | metab_15227 | 0.93345543 | 0.004384 | down |
| Afzelechin 7-apioside | metab_22031 | 0.93049916 | 0.01552 | down |
| 12alpha-Hydroxy-3-oxochola-1,4,6-trien-24-oic Acid | metab_1716 | 0.92958616 | 0.01625 | down |
| Citrulline | metab_23172 | 0.92955921 | 0.04551 | down |
| Polyporusterone E | metab_20327 | 0.92742753 | 0.03565 | down |
| 2-(4-ethyl-3-hydroxyphenyl)-3,4-dihydro-2H-1-benzopyran-3,5,7-triol | metab_12217 | 0.925 | 0.02131 | down |
| 9'-Carboxy-alpha-chromanol | metab_12823 | 0.92491979 | 0.03506 | down |
| 3-Nitrophenol | metab_966 | 0.92453617 | 0.03895 | down |
| 1-Carbapen-2-em-3-carboxylic acid | metab_10737 | 0.92201547 | 0.04243 | down |
| N-Palmitoyl GABA | metab_2441 | 0.92053769 | 0.0179 | down |
| ()11-HDoHE | metab_4441 | 0.91937639 | 0.03847 | down |
| Cytochalasin Npho | metab_1992 | 0.91818717 | 0.01567 | down |
| Demethylated antipyrine | metab_22041 | 0.91794872 | 0.03696 | down |
| (9Z,12Z,14E)-16-Hydroxy-9,12,14-octadecatrienoic acid | metab_9202 | 0.91704669 | 0.04683 | down |
| 3,4-Dihydroxyphenylpropanoate | metab_20913 | 0.91574544 | 0.01042 | down |
| 9-Methylxanthine | metab_11012 | 0.9155496 | 0.02939 | down |
| Calcitroic acid | metab_15597 | 0.91067485 | 0.03093 | down |
| 5-Acetylamino-6-amino-3-methyluracil | metab_11047 | 0.90989353 | 0.04265 | down |
| Kukoamine C | metab_15587 | 0.90865174 | 0.02752 | down |
| Methyl (3x,10R)-dihydroxy-11-dodecene-6,8-diynoate 10-glucoside | metab_21612 | 0.90809026 | 0.009279 | down |
| Goshuyic acid | metab_7116 | 0.90489642 | 0.0327 | down |
| Varanic acid | metab_19534 | 0.90411899 | 0.03305 | down |
| Geniposidic Acid | metab_22662 | 0.90196579 | 0.0384 | down |
| Enokipodin D | metab_1278 | 0.9012118 | 0.01662 | down |
| Gamma-Tocotrienol | metab_16195 | 0.90096694 | 0.02508 | down |
| {[1-(4-methoxyphenyl)pentan-3-yl]oxy}sulfonic acid | metab_21310 | 0.89960159 | 0.04548 | down |
| Deoxyartemsinin | metab_21919 | 0.89878346 | 0.01861 | down |
| [8]-Dehydrogingerdione | metab_12652 | 0.89812195 | 0.00735 | down |
| Yucalexin B'11 | metab_19530 | 0.89591782 | 0.01812 | down |
| 4-Androsten-3,17-dione 19-aldehyde | metab_8684 | 0.89417989 | 0.02956 | down |
| 11-Hydroxyeicosatetraenoate glyceryl ester | metab_15256 | 0.89392696 | 0.03351 | down |
| Pyridinoline | metab_10009 | 0.89163586 | 0.02514 | down |
| 7,8-Dehydro-beta-micropteroxanthin | metab_19376 | 0.89065948 | 0.02707 | down |
| Isothankunic acid | metab_18941 | 0.89031115 | 0.0362 | down |
| D-Urobilin | metab_9548 | 0.88860326 | 0.03597 | down |
| 11'-Carboxy-alpha-chromanol | metab_18618 | 0.88678011 | 0.01008 | down |
| 2-(14,15-Epoxyeicosatrienoyl) Glycerol | metab_20229 | 0.88607311 | 0.01966 | down |
| Gamabufogenin | metab_9190 | 0.88509795 | 0.03933 | down |
| Digoxigenin monodigitoxoside | metab_20314 | 0.88468158 | 0.02033 | down |
| Alectrol | metab_21916 | 0.87864189 | 0.02437 | down |
| 27-Norcholestanehexol | metab_15520 | 0.87804878 | 0.01903 | down |
| Gibberellin A110 | metab_20841 | 0.87708578 | 0.01775 | down |
| (3beta,5alpha,6alpha,7alpha,22E,24R)-5,6-Epoxyergosta-8,14,22-triene-3,7-diol | metab_19169 | 0.87365976 | 0.01285 | down |
| N-Ribosylhistidine | metab_16581 | 0.8725327 | 0.02147 | down |
| Valinopine | metab_3545 | 0.87200179 | 0.01079 | down |
| Yucalexin P8 | metab_13002 | 0.86880072 | 0.001171 | down |
| Resolvin E1 | metab_9926 | 0.86862745 | 0.01251 | down |
| 14-HDoHE | metab_445 | 0.86182723 | 0.01286 | down |
| Unknown 370 | metab_20061 | 0.86034913 | 0.02412 | down |
| Dihydroceramide C2 | metab_152 | 0.85564648 | 0.02094 | down |
| Alpha-Micropteroxanthin B | metab_16081 | 0.85160364 | 0.03046 | down |
| 5,8-Epoxy-5,8-dihydro-10'-apo-b,y-carotene-3,10'-diol | metab_19748 | 0.84932534 | 0.007798 | down |
| Pregnanediol-3-glucuronide | metab_20464 | 0.84918648 | 0.03104 | down |
| Yangonin | metab_12448 | 0.84899683 | 0.01437 | down |
| Isoachifolidiene | metab_12513 | 0.84852217 | 0.04396 | down |
| Ixocarpanolide | metab_15062 | 0.84354839 | 0.0374 | down |
| Desmosflavanone II | metab_10164 | 0.84197394 | 0.01032 | down |
| Beta-Micropteroxanthin | metab_19549 | 0.84102105 | 0.01223 | down |
| 5b-Cyprinol sulfate | metab_15817 | 0.83720374 | 0.02251 | down |
| Oleamide | metab_7144 | 0.83423036 | 0.03777 | down |
| Calcipotriol | metab_15686 | 0.83394754 | 0.03148 | down |
| C-2 Ceramide | metab_7566 | 0.82962677 | 0.01789 | down |
| N,N-Dimethylsphingosine | metab_18736 | 0.82804115 | 0.02084 | down |
| Pubescenol | metab_20054 | 0.81070323 | 0.02759 | down |
| 4-ethylamino-6-isopropylamino-1,3,5-triazin-2-ol | metab_19265 | 0.79975186 | 0.02896 | down |
| Schidigeragenin B | metab_20596 | 0.79254202 | 0.03056 | down |
| Goshonoside F1 | metab_16088 | 0.77535739 | 0.01436 | down |
| 6-Deoxocastasterone | metab_19655 | 0.7725796 | 0.02495 | down |
| Nootkatol | metab_15545 | 0.76811997 | 0.04036 | down |
| 3,5,9-Trihydroxyergost-7-en-6-one | metab_16100 | 0.76038781 | 0.04578 | down |
| 11-Dihydro-12-norneoquassin | metab_20378 | 0.74700762 | 0.03577 | down |
| 1-a,24R,25-Trihydroxyvitamin D2 | metab_19798 | 0.74604334 | 0.003996 | down |
| Erinapyrone A | metab_8704 | 0.72891727 | 0.01295 | down |
| Withanolide | metab_15222 | 0.71816284 | 0.03008 | down |
| 4a-Methylzymosterol-4-carboxylic acid | metab_7301 | 0.45565272 | 0.0314 | down |
